# Interprovincial Differences in Access to Phosphate Lowering Medication; Implications for Care as Canada Moves Toward a National Pharmacare Program

**DOI:** 10.1177/20543581231207467

**Published:** 2023-11-10

**Authors:** Tristin E. Wilson, Rachel M. Holden

**Affiliations:** 1 Department of Medicine, Queen’s University, Kingston, ON, Canada

**Keywords:** CKD (chronic kidney disease), phosphate, pharmacare, phosphate binders, kidney replacement therapy

Prescribed medication represents the second biggest expenditure in health care, after hospitals. However, Canada is the only country in the world with a public health care system where citizens do not have uniform access to publicly funded medication insurance.^1^ As a result, there is a patchwork of more than 100 government-run drug insurance programs where the coverage provided, and to whom it is provided, varies widely. For patients with end stage kidney disease (ESKD), access to medications for the management of chronic kidney disease-mineral bone disorder (CKD-MBD) is one example where substantial interprovincial differences exist in access to potentially safer but more expensive medications for lowering phosphate levels.

Approximately 1 in 5 Canadians do not have medication insurance or have inadequate coverage to meet their needs.^2^ The most vulnerable groups include ethnic minorities, immigrants, people with low incomes, women, and younger people who are more likely to work in part-time or contract positions without employer benefits. In the 2022 budget, the Government of Canada announced their commitment to passing a Canada Pharmacare Act by 2023. What will this mean for publicly funded access to medications such as non-calcium based phosphate binders (NCBPBs) where current access ranges from no access in provinces such as Alberta to fairly liberal access in provinces such as Quebec and for recipients of nationally funded programs?

**Figure 1.**
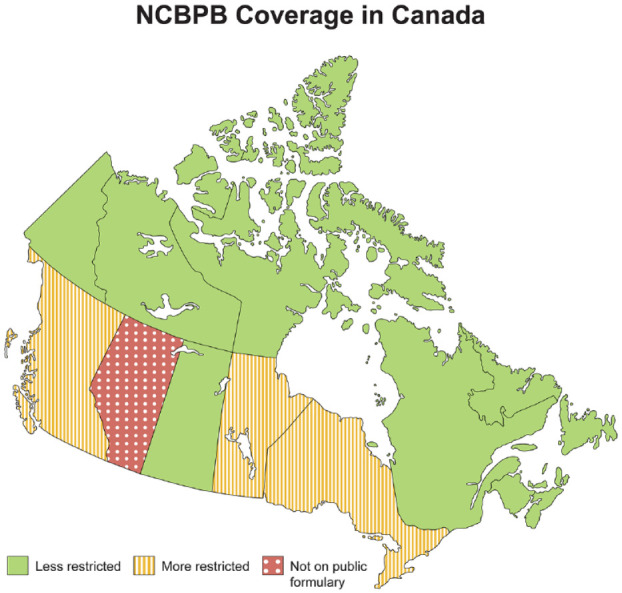
NCBPB coverage map. *Note.* NCBPB = non-calcium based phosphate binders.

For patients with ESKD who require kidney replacement therapy (KRT), the most common cause of morbidity and mortality is cardiovascular disease (CVD).^3^ This is due, in part, to the alterations in mineral metabolism that affect the cardiovascular and skeletal systems. ^4^ The Kidney Disease Improving Global Outcomes (KDIGO) clinical practice guidelines and the Canadian Society of Nephrology (CSN) working group suggest that serum phosphate levels should be “lowered towards the normal range” in patients receiving KRT.^4,5^ Although diet and dialytic phosphate removal are important, the majority of patients receiving KRT in Canada take phosphate binding medication to achieve this goal.^6^ Calcium-based phosphate binders (CBPBs) and NCBPBs are both effective in lowering serum phosphate. When directly compared, NCBPBs are more expensive^7^ but expose patients to less calcium, decrease the progression of vascular calcification,^8^ and are not associated with hypercalcemia.^9^ Based on these risks, the KDIGO clinical practice guidelines suggest restricting the dose of CBPBs to 1500 mg or less of elemental calcium per day.^4^ The CSN working group acknowledges the potential for excessive calcium intake to exacerbate vascular calcification. However, a precise ceiling for total calcium intake has not been identified given that the benefits of using NCBPBs were suggested in studies measuring the severity of arterial calcification but not subsequently supported by large randomized controlled trials (RCTs) that examined clinical outcomes.^10,11^ These include The Dialysis Clinical Outcomes Revisited (DCOR) trial (Sevelamer)^12^ and the Landmark trial (Lanthanum) that reported on hospitalizations and mortality.^13^ The CSN working group recognizes that avoiding calcium might benefit individual patients (ie, those with concomitant hyperphosphatemia and hypercalcemia), although the added cost of NCBPBs makes it difficult to support widespread use without clear evidence of benefit and cost-effectiveness.^14^ In real-world practice, multiple factors influence the choice of phosphate binder including phosphate-lowering effectiveness, calcium exposure, pill burden, side effects, and cost-effectiveness. However, in many cases, the decision is driven by private drug benefit coverage and, for many, the province where the patient resides (Figure 1).

Once Health Canada approves a drug for use, the country’s public drug programs decide if the drug will be eligible for public reimbursement. The Canadian Agency for Drugs and Technologies in Health (CADTH) uses objective evidence to provide non-binding reimbursement recommendations to federal, provincial, and territorial public drug plans (with the exception of Quebec). In 2009, the CADTH concluded that the routine use of sevelamer or lanthanum was not supported by the current literature.^15^ Furthermore, it was recommended that drug plans consider a drug class review of phosphate binders given the differential cost of these agents and the results of RCTs that failed to demonstrate an effect of NCBPBs on morbidity and mortality.

Drug reimbursement programs have responded differently to the same evidence and non-binding recommendations; therefore, it is likely that the lack of high-quality evidence has led to eligibility criteria based on opinions that differ between experts by province or jurisdiction (Table 1). In some provinces (eg, Saskatchewan, Quebec and Atlantic provinces), NCBPBs are covered if calcium is “inappropriate or not tolerated” or “contraindicated or sub-optimal at controlling phosphate levels.” Other provinces have placed greater restrictions (Ontario and Manitoba), and finally, residents of Alberta must cover the cost personally. Qualified Veterans or clients of Correctional Services may receive an NCBPB as a standard benefit. Members of the Canadian Armed Forces and First Nations and Inuit may qualify if phosphate levels are not well-controlled with calcium despite restriction of dietary phosphate, modification of dialysate calcium, or discontinuation of CBPBs and vitamin D analogues. To date, Manitoba is the only province to identify a ceiling limit for calcium intake (>4500 mg of elemental calcium per day), a value that exceeds the KDIGO recommendation by 3-fold. RCTs have demonstrated that NCBPBs attenuate the progression of coronary artery calcification (CAC), a surrogate measure of CVD, compared with CBPBs.^8-10^ Ontario’s Exceptional Access Program will consider coverage for patients with elevated phosphate levels who have incidental CAC observed on CT; however, the intention is not to use this modality to screen patients. In Newfoundland and Labrador, Nova Scotia, Prince Edward Island, Manitoba, and Ontario, NCBPBs are covered for patients who develop calciphylaxis, a rare but highly morbid condition. Duration of coverage also varies by province.

**Table 1.** Non-Calcium Based Phosphate Binder Coverage Criteria by Canadian Population.

Population	Program	Drug	Coverage requirements	Approval duration
Alberta	N/A	None	N/A	
British Columbia	Renal Dialysis Formulary	Sevelamer^a^ Lanthanum	Ca > 2.6 mmol/L (sustained), AND inadequate control of Ph, Ca, and PTH with a CBPB	Determined by renal team
Manitoba	Exceptional Drug Status	Sevelamer^a^	Ph >1.8 mmol/L, AND Ca > 2.65 mmol/L despite reduction in calcium dosage, OR Soft tissue calcification, OR CBPB not appropriate or tolerated, OR Patients requiring >4500 mg Ca per day	Individual basis
New Brunswick	Drug Plans Formulary	Sevelamer Lanthanum Sucroferric Oxyhydroxide	For Lanthanum and Sucroferric Oxyhydroxide: Ph >1.8 mmol/L, AND For Lanthanum only: CBPB are not appropriate or tolerated, OR Inadequate control of Ph with CBPB	Long-term Lanthanum only: Initial: 6 months Renewal: long-term with improved Ph levels
Newfoundland and Labrador	Special Authorization	Sevelamer Sucroferric Oxyhydroxide	Ph >1.8 mmol/L, AND Hypercalcemia (corrected), OR Calciphylaxis, OR Inadequate control of Ph with CBPB	Initial: 6 months (1 yr for sevelamer carbonate) Renewal: 1 yr with improved Ph levels
Nova Scotia	Exceptional Status Drugs	Sevelamer^a^ Sucroferric Oxyhydroxide	Ph >1.8 mmol/L, AND Hypercalcemia (corrected), OR Calciphylaxis, OR Inadequate control of Ph with CBPB	Initial: 6 months Renewal: 1 yr with improved Ph levels
Ontario	Exceptional Access Program	Sevelamer Lanthanum Sucroferric Oxyhydroxide	Ph > 1.8 mmol/L (sustained) AND Ca >2.65 mmol/L (corrected, sustained), AND Calciphylaxis, OR CAC, OR Sucroferric Oxyhydroxide only: Other types of calcifications on case-by-case basis	Lifetime
Prince Edward Island	Pharmacare Formulary	Sevelamer	Ph > 1.8 mmol/L, AND Hypercalcemia (corrected), OR Calciphylaxis, OR Inadequate control of Ph with CBPB	Initial: 6 months Renewal: 1 year with improved phosphate levels
Quebec	Exceptional Medications	Sevelamer Lanthanum Sucroferric Oxyhydroxide	CBPB are not appropriate or tolerated, OR Inadequate control of Ph with CBPB	No maximum period
Saskatchewan	Exceptional Drug Status Program	Sevelamer Lanthanum Sucroferric Oxyhydroxide	CBPB are not appropriate or tolerated, OR Inadequate control of Ph with CBPB	Individual basis
Canadian Armed Forces	Drug Benefit Plan	Sevelamer^b^	Elevated Ph, OR Elevated Ph x Ca product despite dietary Ph restriction and CBPB use, OR Elevated Ca levels despite reduction in Ca dosage, OR Normal-high Ca with PTH <10.6 pmol/L	Not listed
Correctional Services Canada Offenders	Correctional Services Canada National Formulary	Sevelamer^b^ Lanthanum	Dialysis patient	Indefinite
First Nations and Inuit	Non-Insured Health Benefits Program	Sevelamer^a^	Elevated Ph, OR Elevated Ph × Ca product despite dietary Ph restriction and CBPB use, OR Elevated Ca levels despite discontinuation of CBPB, OR Normal-high Ca with PTH <10.6 pmol/L	Prior approval required
Qualified Veterans	Veterans Affairs Canada Program	Sevelamer^b^ Sucroferric Oxyhydroxide	N/A	Standard benefit

*Note.* Hypercalcemia (corrected) indicates Ca must be corrected for albumin. Ca = serum calcium; Ph = serum phosphate; PTH = serum parathyroid hormone; CAC = coronary artery calcification; CBPB = calcium-based phosphate binder. Yr = year.

a Sevelamer carbonate only.

b Sevelamer HCL only.

CSN policy states that accessibility to high-quality kidney care should be uniform across all provinces, yet differences in eligibility criteria seemingly do not allow for equitable access.^16^ By way of example, a 66-year-old female receiving dialysis could receive treatment with an NCBPB if she is a veteran or a client of Correctional Services. If she doesn’t tolerate calcium, she could receive an NCBPB if she is Inuit or First Nations, an active member of the Canadian Armed Forces or if she lives in Quebec or Saskatchewan (Table 1). If she has hypercalcemia, she qualifies if she lives in Newfoundland and Labrador or British Columbia but she would also require elevated phosphate levels if she lives in Ontario, New Brunswick, Manitoba, Prince Edward Island, or Nova Scotia. If she has calciphylaxis, she would qualify if she lives in Ontario, Newfoundland and Labrador, Prince Edward Island, or Nova Scotia. In Ontario, she qualifies if she has evidence of CAC demonstrated incidentally either by an expensive CT imaging test (exposing her to 2-5 millisieverts of radiation) or by an angiogram.

Patients with greater access to NCBPBs may benefit from improved management of mineral metabolism parameters. In an analysis of Dialysis Outcomes and Practice Patterns Study II (DOPPS II) data, patients living in provinces with less-restricted access to NCBPBs were less likely to be receiving calcium and more likely to be receiving an NCBPB and have mineral metabolism parameters within the target range.^17^ Whether these national and provincial differences in accessibility also impact clinical outcomes is unknown.

The pan-Canadian Advisory Panel on a Framework for a Prescription Drug List was tasked, in a 3-stage process, with establishing a set of principles to guide the selection and management of proposed drugs and related products for a pan-Canadian formulary.^18^ The 6 guiding principles include: universal and integrated; equitable; effective, safe and high quality; sustainable; efficient and timely; and inclusive, transparent with fair process. As part of stage 1, the panel provided nonbinding recommendations for specific drug inclusions in 3 therapeutic areas (cardiovascular disease, diabetes, and mental health) that together account for approximately 62% of prescriptions. The panel specifically chose not to build on existing public formularies because of known gaps between the different formularies, including the significant differences with respect to who may be eligible for coverage. The report, published in June 2022, considered online consultation acquired from feedback from key stakeholders and health system partners.^18^ Several topics were acknowledged to be beyond the scope of this initial report including assessment of current drug plan processes and expectations about whether or how coverage on existing plans might be impacted by a pan-Canadian formulary. This latter issue, clearly relevant for the discussion at hand, requires clarification prior to stage 2 as the process is applied to the 11 other therapeutic areas.

The second-stage process inevitably will create tension as decision-making expands to more specialized therapeutic areas. Arguably, the most defensible process is inclusion of products that add value compared with the alternatives or standard of care currently being provided. But how is value defined? Should the value of a specific drug be viewed from the perspective of its impact on the population overall? If so, therapeutics for chronic diseases with much greater prevalence than ESKD could be advantaged. How will we balance the values of all Canadians with the values of patients and caregivers who are directly affected by the decisions and what is the role of specific advocacy groups in this process? Value from an economic perspective could be considered as the incremental net benefit (INB) of one treatment over another. A recent systematic review and meta-analysis examined the INB of phosphate binders under current practice guidelines for the treatment of elevated phosphate levels.^7^ In dialysis patients, compared with CBPBs, use of either sevelamer or lanthanum as first-line treatment was not found to be cost-effective. Disease-specific stakeholder consultation will be part of the second stage and will be one opportunity for our societies, kidney care providers and patients/caregivers to provide their opinions and values not only for the CKD-MBD realm but for other therapeutic areas within Nephrology. Taken together, this process is one of difficult issues and difficult choices but the demand for health care is greater than the public health care system’s ability to meet that demand.

The panel emphasized that continuity of care would be critical. All therapies included in current drug plans that are not included in the pan-Canadian formulary *could* continue to remain available through those plans; however, how this would be funded and who the payer of last resort would require further consultation. In this process, it will be important to avoid introducing additional logistical and administrative barriers. It is also important to consider whether reduced access to these treatments in the future will lead to patient and physician distress. Should a change occur in how drugs are reimbursed, the panel suggested putting measures in place so that patients are safely transitioned to the included drug. It is not explicitly stated what these measures should be. Literature on de-prescribing suggests that a majority of older patients are willing to de-prescribe if they have a good relationship with the recommending caregiver, highlighting the importance of communication and care continuity. Barriers to de-prescribing exist when patients feel well on their current medicines, when they are convinced that they need all their medicines and when they fear negative consequences.^19^ How strongly patients feel about calcium avoidance remains to be seen. Physicians also fear de-prescribing if it will result in negative patient outcomes or upset patients and/or family members.^20^ Without careful consideration of all these issues, patients may feel like the system has abandoned them with the goal of saving money rather than improving their outcomes.

As the national pharmacare program inevitably unfolds, and the decision regarding whether to universally fund NCBPBs is made, it is imperative that we proactively address the reality that negative trial results render many of our clinical practice guidelines regarding drug therapies as “suggestions.” Although high phosphate levels are a risk factor for poor clinical outcomes in patients with ESKD, no trial to date has been conducted to show that lowering phosphate is beneficial. This reality represents a major conundrum that affects daily practice driven, in part, by strongly held assumptions. First, that tight phosphate control will attenuate the adverse effects of hyperphosphatemia, and second, that excessive calcium loading over time is undesirable. We have embraced opinion based practice guidelines that recommend lowering phosphate and, as a result, more than 80% of patients take phosphate binders accounting for up to 30% of daily pill burden. However, clinical practice uncertainties are frequently encountered in nephrology because few trials have been conducted that examine clinical outcomes and the large RCTs that have been done yield negative results. The KDIGO Controversies Conference on challenges in conducting clinical trials in nephrology suggested that the trials conducted in patients with kidney disease have been too small to detect treatment effects of a magnitude that would be realistic to achieve with a single intervention, such as phosphate lowering.^21^ This siloing is true for the CKD-MBD realm where the large trials have not tested multiple interventions that target different aspects of the interrelated triad of laboratory abnormalities, bone disease and vascular calcification. As a result, the commonly held assumptions previously described have been challenged and/or refuted by the results of trials designed to alter a single parameter and that address major outcomes such as mortality for which the cause may be difficult to adjudicate and may not be sensitive to phosphate lowering. Finally, the benefits of NCBPBs for many patients may be truly equivocal because of limited life span expectancy.

If a national pharmacare program chooses to limit access to these drugs, then an approach to who should qualify will be both a necessary step and a challenge. Two important large-scale trials are currently in process. The PHOSPHATE and HiLo trials both address the critical issue of phosphate targets in patients receiving dialysis however the results from these trials will likely not be available to inform the initial decision unless there are substantial delays in the process.^22,23^ Until then, it is reasonable that patients receiving dialysis with elevated calcium levels and sustained hyperphosphatemia despite reduction in calcium and active vitamin D, be considered for coverage for NCBPBs. However, it is also reasonable that effectiveness of the therapy be demonstrated for on-going coverage. An alternative, but unstudied, viewpoint is that it may be the patient with well-managed dietary intake and controlled phosphate levels who would most benefit over the long-term from a reduction in their calcium burden. At present, this patient would not qualify for a medication to maintain their phosphate control and achieve this goal raising the provocative question as to whether existing reimbursement programs are biased against “healthy adherers.” Calciphylaxis is rare, but mortality approaches 50%, with little by way of RCT evidence to guide management.^24^ Many practicing kidney caregivers would wish to avoid excessive calcium and active vitamin D exposure in these patients for the best chance of surviving their condition. The approach in Ontario where coverage is provided to patients with elevated phosphate levels who also have coronary artery calcification acknowledges the trial evidence that NCBPBs attenuate progression of calcification but as an approach requires re-examination for the following reasons: the intent is not to use this expensive imaging test to screen for CAC, it fails to consider that vascular calcification is a diffuse process that is not limited to the coronary arteries and leads to other important clinical outcomes such as heart failure and amputation, and finally there are more widely available, less expensive imaging modalities to detect calcification such as lateral spine X-rays or echocardiograms.

With the development of a National Pharmacare program for Canada, the qualifying criteria for NCBPB coverage must be determined using the best evidence available with the goal of providing the highest quality of kidney care to Canadians uniformly across provinces and jurisdictions. A difficult challenge lies ahead as strongly held assumptions about the harm associated with calcium loading have been refuted by the negative results of trials addressing a single parameter within a complex and multi-system disease. With the utmost objectivity, unencumbered by industry and other influences, we must strive responsibly to achieve the best possible outcomes for Canadians living with kidney disease.
